# Label-free mid-infrared dichroism-sensitive photoacoustic microscopy for histostructural analysis of engineered heart tissues

**DOI:** 10.1038/s41377-025-02117-0

**Published:** 2026-01-04

**Authors:** Eunwoo Park, Dong Gyu Hwang, Hwanyong Choi, Donggyu Kim, Joongho Ahn, Yong-Jae Lee, Tae Joong Eom, Jinah Jang, Chulhong Kim

**Affiliations:** 1 Department of Convergence IT Engineering, Pohang University of Science and Technology (POSTECH), Pohang, Republic of Korea; 2 Medical Device Innovation Center, Pohang University of Science and Technology (POSTECH), Pohang, Republic of Korea; 3 Center for 3D Organ Printing and Stem Cells, Pohang University of Science and Technology (POSTECH), Pohang, Republic of Korea; 4 Department of Mechanical Engineering, Pohang University of Science and Technology (POSTECH), Pohang, Republic of Korea; 5 Department of Electrical Engineering, Pohang University of Science and Technology (POSTECH), Pohang, Republic of Korea; 6 Opticho Inc., Pohang, Republic of Korea; 7 Engineering Research Center for Color-modulated Extra-sensory Perception Technology, Pusan National University, Busan, Republic of Korea; 8 Department of Congo-Mechatronics Engineering & Optics and Mechatronics Engineering, Pusan National University, Busan, Republic of Korea; 9 Department of Medical Science and Engineering, Pohang University of Science and Technology (POSTECH), Pohang, Republic of Korea; 10 Institute for Convergence Research and Education in Advanced Technology, Yonsei University, Seoul, Republic of Korea; 11 Graduate School of Artificial Intelligence, Pohang University of Science and Technology (POSTECH), Pohang, Republic of Korea

**Keywords:** Imaging and sensing, Microscopy

## Abstract

Many biological tissues, such as cardiac muscle, tendons, and the cornea, exhibit highly organized microstructural alignment that is critical for mechanical and physiological functions. Disruptions in this structural organization are commonly associated with pathological conditions such as fibrosis, infarction, and cancer. However, conventional histological imaging techniques rely on immunofluorescence or histochemical staining, and they evaluate tissue alignment via non-physical 2D gradient-based calculation, which is labor-intensive, antibody-dependent, and prone to variability. Here, we demonstrate label-free mid-infrared dichroism-sensitive photoacoustic microscopy (MIR-DS-PAM), an analytical imaging system for cardiac tissue assessments. By combining molecular specificity with polarization sensitivity, this method selectively visualizes protein-rich engineered heart tissue (EHT) and quantifies the extracellular matrix (ECM) alignment without any labeling. The extracted dichroism-sensitive parameters, such as the degree of dichroism and the orientation angle, enable histostructural evaluation of tissue integrity and reveal diagnostic cues in fibrotic EHT. This technique offers a label-free analytical tool for fibrosis research and tissue engineering applications.

## Abstract (web summary)

Mid-infrared dichroism-sensitive photoacoustic microscopy enables label-free, quantitative histostructural analysis by combining spectral specificity and polarization sensitivity to visualize protein-rich components and evaluate anisotropic tissue alignment.

## Graphical abstract

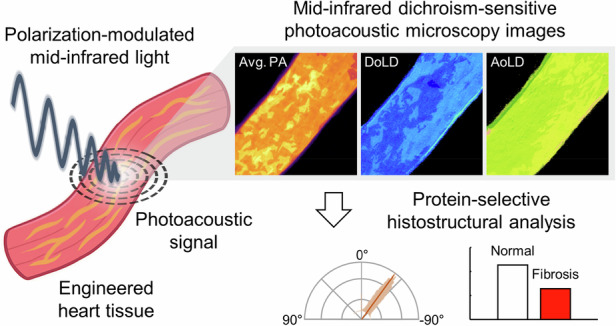

## Introduction

Many tissues in the human body—such as cardiac and skeletal muscles, tendons, and the cornea—exhibit highly organized structural arrangements essential for their function^1–3^. The spatial organization of microstructures, including the alignment of cellular components and the extracellular matrix (ECM), plays a crucial role in determining a tissue’s mechanical properties, physiological function, and pathological transitions. Therefore, a quantitative assessment of alignment can be valuable in diagnosing disease, monitoring therapeutic responses, and evaluating the efficacy of tissue engineering models^4,5^. For example, disruptions in cardiac tissue orientation can lead to or reflect such pathological changes as myocardial infarction and hypertrophy, while collagen alignment is a hallmark of fibrosis and tumor progression^6,7^. Tissue alignment is typically assessed using histological imaging techniques, such as immunofluorescence or hematoxylin and eosin (H&E) staining^8,9^. However, these methods are labor-intensive, antibody-dependent, and prone to variability depending on imaging conditions or staining expression, limiting an objective assessment. Moreover, alignment quantification in techniques like confocal fluorescence microscopy (CFM) relies on indirect and non-physical estimation based on 2D gradient calculations in certain fluorophore-expressed images. Consequently, there is a clear need for label-free imaging, quantitative analysis, and objective assessment of tissue structures^10^.

Photoacoustic (PA) microscopy (PAM) is an alternative imaging modality to light microscopy^11–13^. Based on the light absorption of endogenous chromophores, PAM can provide structural and functional information in label-free tissue images^14,15^. By employing the optimal optical wavelength for the specific biomolecules, high-contrast tissue imaging can be achieved for each application^16–19^. In histopathological applications, PAM has primarily used ultraviolet (UV) excitation to highlight cell nuclei^20,21^. UV-PAM enables high-resolution histological evaluation and cancer diagnosis in multiple organs. While UV-PAM is effective for nuclear imaging, it lacks chemical specificity and functional analysis. In contrast, mid-infrared (MIR)-PAM enables analytical histology with the benefit of high spectral resolution^22^. While Fourier transform infrared (FTIR) technology spectroscopically identifies molecular components, MIR-PAM provides bond-selective imaging of proteins, lipids, and carbohydrates by using functional group and fingerprint optical regions in MIR^23–25^. Furthermore, dichroism-sensitive (DS)-PAM has been developed, enhancing PAM’s functional imaging capability with polarization contrast^26–28^. The macromolecular alignment in birefringent tissues determines their optic axes, which create an anisotropic interaction of light and biomolecules. Capitalizing on this anisotropic interaction, various label-free imaging modalities have been utilized to investigate structural features within the tissue. For example, polarization-sensitive optical coherence tomography (PS-OCT) examines backscattered light with rotated polarization states^29–31^. PS-OCT quantitatively analyzes tissue’s birefringence via the phase retardation, degree of polarization uniformity, and optic axis, but it lacks molecular selectivity. Moreover, its sensitivity in ultrathin tissue is fundamentally limited by an insufficiently long optical path length to induce measurable phase retardation (Fig. S1). Second harmonic generation (SHG) microscopy, a high-resolution quantitative technique based on the nonlinear optical effect^32–34^, is highly sensitive to non-centrosymmetric molecules like collagen, but it has limited penetration depth. In contrast, depending on the anisotropic optical absorption, DS-PAM selectively detects PA signals and simultaneously characterizes the DS parameters, i.e., the degree of dichroism and the orientation angle^35,36^. Tissue polarimetry using DS-PAM provides a quantitative assessment of fibrous tissues with high birefringence^37,38^. The DS parameters correlate with the collagen fiber density and orientation, which are crucial diagnostic markers for assessing fibrotic disease and myocardial remodeling.

In this work, we present label-free DS-PAM for assessing tissue development and disease modeling. Combining protein-selective MIR-PAM with DS functionality, dual-contrast MIR-DS-PAM can simultaneously quantify a tissue’s protein content and image its microstructural organization. Specifically, MIR-DS-PAM selectively images protein components within the ECM, followed by polarization analysis to extract fiber alignment characteristics such as orientation and uniformity. To validate this approach, we fabricated an engineered cardiac tissue (EHT)—in which alignment serves as a critical functional indicator—and we induced pathological conditions for evaluation. The results were consistent with those obtained using CFM, confirming the reliability of MIR-DS-PAM. As the tissue matured, we observed increased protein accumulation and more uniform fiber alignment. In contrast, under fibrotic conditions, we detected changes in protein content and a marked reduction in alignment uniformity, indicating a disorganized and heterogeneous tissue structure. These findings demonstrate that MIR-DS-PAM enables label-free monitoring of both tissue development and fibrotic remodeling, highlighting its potential as a functional imaging tool not only for cardiac tissue assessment but also for broader applications in fibrosis evaluation and tissue engineering research.

## Results

### MIR-DS-PAM

**Fig. 1:**
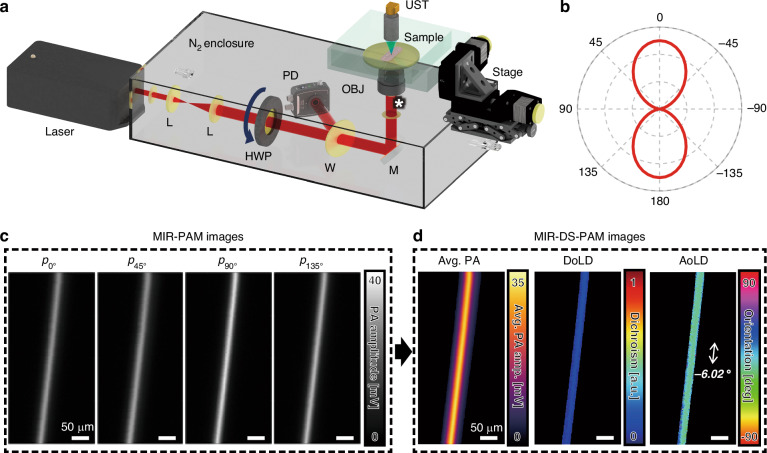
MIR-DS-PAM imaging system. **a** System schematic of MIR-DS-PAM. **b** Angle dependency of the laser beam. **c** MIR-PAM maximum amplitude projection (MAP) images arranged by polarization states. **d** MIR-DS-PAM images: average MAP, DoLD, and AoLD, respectively. Scale bars, 50 μm. L lens, HWP half-wave plate, W window, PD photodiode, M mirror, OBJ, objective lens, UST ultrasound transducer

We constructed the MIR-DS-PAM system illustrated in Fig. 1a. The MIR light source was a pulsed quantum cascade laser (QCL), set to a center wavelength of 6.0 μm to correspond with an absorption peak in the amide I band (C = O stretching vibration). Passing through an enclosure filled with N_2_ to minimize optical loss, the laser beam was expanded, collimated, and linearly polarized. The polarized light, modulated by a half-wave plate (HWP), illuminated the target tissue. Confirming the polarization’s linearity, Fig. 1b shows the initial polarization state of the light, measured at the position marked by a white asterisk below the objective (OBJ) in Fig. 1a. The measured lateral resolution was about 6.6 μm for all polarization states, and the measured tissue penetration depth was at least 60.7 μm^25^.

The DS-PAM functionality was validated by imaging a 20 μm-diameter polyamide monofilament placed on a ZnSe window. Figure 1c shows PA maximum amplitude projections (MAPs) for four incident polarization angles. Because the thread mimics a fibrous structure, the PA signal amplitude varies depending on the dichroic properties. The acquired PA data were used to calculate and map the average PA MAP, the degree of linear dichroism (DoLD), and the orientation angle of linear dichroism (AoLD). DoLD and AoLD were derived from the Stokes parameter as follows^35^:

1 $${\rm{DoLD}}=\frac{\sqrt{{Q}_{{PA}}^{2}+{U}_{{PA}}^{2}}}{{I}_{{PA}}}$$

2 $${\rm{AoLD}}=\frac{1}{2}{\tan }^{-1}\left(\frac{{U}_{{PA}}}{{Q}_{{PA}}}\right)$$

Here, ${I}_{{PA}}={p}_{0^\circ }+{p}_{90^\circ }$, ${Q}_{{PA}}={p}_{0^\circ }-{p}_{90^\circ }$, and ${U}_{{PA}}={p}_{45^\circ }-{p}_{135^\circ }$. ${p}_{\theta }$ represents PA data at polarization angle $\theta$ of the incident light. Figure 1d presents MIR-DS-PAM images of the average PA MAP, the DoLD, and the AoLD. The average PA MAP was reconstructed equivalently to the MIR-PAM image. The overall DoLD and AoLD were measured consistently along the longitudinal direction as 0.21 ± 0.06 and −6.02° ± 4.29°, respectively. The MIR-DS-PAM system mapped the birefringent characteristics of threads well (Fig. S2), successfully demonstrating its DS functionality for fibrous samples.

### Label-free EHT section imaging

**Fig. 2:**
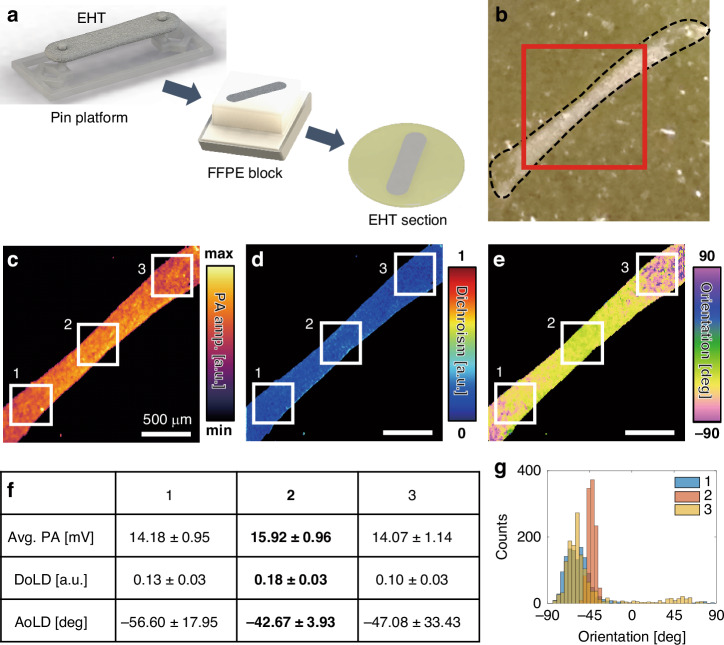
Label-free EHT imaging using MIR-DS-PAM. **a** Preparation of an EHT section. **b** Photograph of the EHT slide. The inset black dashed line and red box indicate the EHT boundary and imaging area, respectively. **c** Average PA maximum amplitude projection. **d** DoLD map. **e** AoLD map. Scale bars, 500 μm. **f** Quantitative comparison of DS parameters in the regions of interest (ROIs) indicated by the white boxes in (**c**–**e**), respectively. Data are presented as mean ± standard deviation. **g** Histograms of AoLD in the ROIs. EHT engineered heart tissue, FFPE formalin-fixed paraffin-embedded

To validate the feasibility of MIR-DS-PAM for EHT assessments, we followed the process shown in Fig. 2a to prepare a formalin-fixed paraffin-embedded (FFPE) EHT section slide. Bioprinted EHT was grown on a pin platform until maturation (details are in “Methods”). After the manufactured EHT was processed into the FFPE block, an unlabeled tissue section was placed on a ZnSe window (Fig. 2b), chosen for its high transmission of MIR light. PA images were obtained at four incident angles in the imaging area indicated by the red box in Fig. 2b. By calculating the MIR-DS-PAM images of label-free EHT according to Eqs. 1 and 2, the average PA MAP, DoLD, and AoLD are presented in Fig. 2c–e, respectively. Because the ECM contains many fibrous proteins, such as collagen and fibronectin^39^, the PA MAP visualizes the protein-rich EHT, and the DS parameters describe the ECM alignment.

We further examined the EHT around the pins of the platform at both ends (points 1 and 3) and at the center (point 2), as indicated by the white boxes (Fig. 2f). The highest PA signal amplitude is at point 2, implying the protein concentration is highest there. Among the DS parameters, DoLD shows the highest value, which implies that the birefringent composition is dense. Notably, the AoLD uniformity is higher at point 2 than at points 1 and 3, suggesting that the fibrous structure of the ECM is well aligned at the center. Near the pins, ECM tends to align circumferentially due to localized mechanical constraints imposed by the anchoring structure. This results in lower orientation uniformity compared to the central region, where longitudinal tension promotes unidirectional fiber alignment^9^. In particular, the dispersed nature of alignment near the pins can result in AoLD values differing by up to 90 degrees across selected regions of interest, and the spatial trend exhibits gradually increasing alignment uniformity toward the center. Figure 2g shows a histogram of AoLD for each region of interest. While the ECM becomes loosely tangled near the pins, the center is tightly aligned with abundant fibrous proteins, which can represent the characteristics of the corresponding EHT.

### EHT integrity assessment

**Fig. 3:**
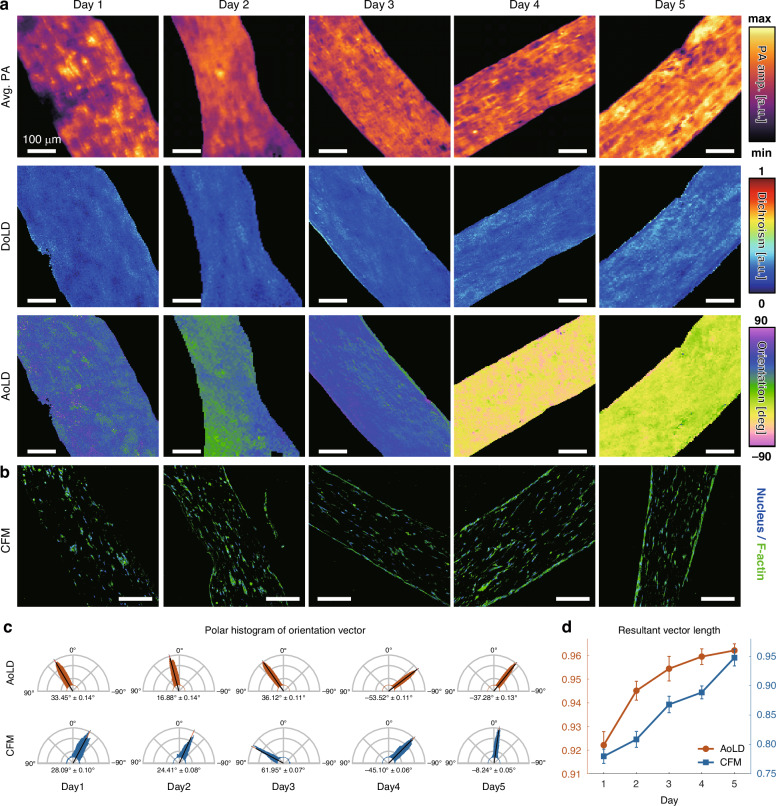
EHT integrity assessment with maturation. **a** MIR-DS-PAM and (**b**), CFM images by the day. Scale bars, 100 μm. **c** Polar histograms of the orientation vectors in each sample. Average angles are presented as mean ± 95% confidence interval. **d** Comparison of the resultant vector lengths by the day (*n* = 3, mean ± standard deviation)

We used MIR-DS-PAM to qualitatively assess EHT’s integrity as it matured. EHT specimens were cultured and fixed at sequential time points from days 1 to 5. Figure 3a presents label-free MIR-DS-PAM images of FFPE EHT sections (Table S1). As part of a systematic acquisition strategy, PA images were acquired from 5 μm-thick non-deparaffinized tissue sections and normalized pixel-by-pixel using the corresponding laser fluence for each polarization state. However, the average PA amplitude exhibits heterogeneity both across and within samples, which may be attributed to variations in cellular organization and local protein density during EHT maturation. Although conventional MIR-PAM enables label-free visualization of the ECM, it is insufficient for robust evaluation of the EHT’s structural integrity. Analysis based on DS parameters provided more insightful information. The DoLD increases gradually over time, suggesting EHT reconstruction, but there are no dramatic changes because the composition of the ECM does not change significantly. The AoLD effectively reflects the orientations of the EHT (Fig. S3). Notably, the standard deviation of AoLD progressively decreases, indicating that the ECM fibers became increasingly aligned over time—consistent with the microstructural alignment of the EHT. This indication is supported by corresponding immunofluorescence-stained CFM images that use specific dyes for each biomarker (Fig. 3b). As represented by F-actin, a major cytoskeletal component for maintaining structural and functional properties of heart tissue, cellular alignment and structural arrangement become apparent in the EHT.

To quantitatively assess the EHT’s integrity over time, we measured the resultant vectors in the orientation (Fig. 3c and Fig. S3). The average angles of the resultant vectors reflect the predominant ECM orientation, which corresponds well with the azimuthal positioning of the sample on the imaging window. Furthermore, the resultant vector lengths, which indicate orientation uniformity, were increased by the day in both modalities, implying that the ECM alignment becomes robust with maturation (Fig. 3d). In CFM images, structural information is determined by calculating the gradient between adjacent pixels from only the expressed immunofluorescence signals, but this does not sufficiently reflect the various ECM components within the EHT, and non-physical calculations indirectly interpret the characteristics. On the other hand, MIR-DS-PAM provides a comprehensive assessment of the EHT by quantifying the intrinsic physical properties of the overall ECM, enabling consistent and reliable histostructural analysis without any labeling.

### MIR-DS-PAM in fibrotic EHT

We applied MIR-DS-PAM histological analysis to the EHT fibrosis model. Because cardiac fibrosis is characterized by fibroblast overproliferation, myofibroblast activation, and ECM accumulation that causes tissue dysfunction and disorganization, a comprehensive ECM assessment is 1`essential to interpret diagnostic cues^40,41^. We investigated two types of EHT fibrosis: cell ratio^41–43^ and drug-induced fibrosis^44–48^.

**Fig. 4:**
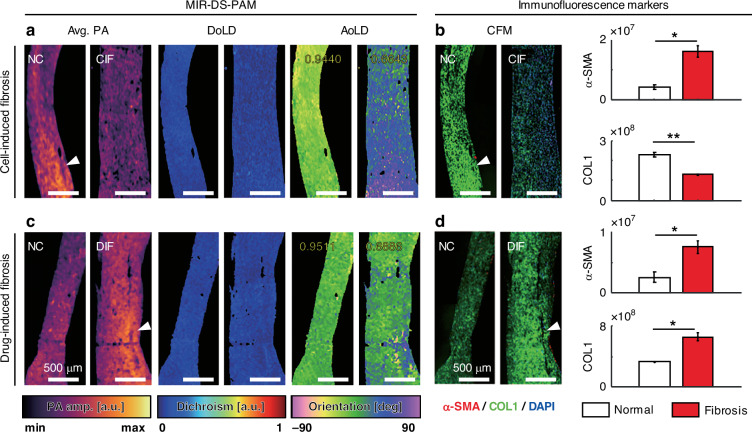
MIR-DS-PAM in EHT fibrosis. **a** MIR-DS-PAM images of cell-induced fibrosis (CIF); **b** the corresponding immunofluorescence analysis (*n* = 2, mean ± standard deviation, *p < 0.05, **p < 0.01 by t-test). **c** MIR-DS-PAM images of drug-induced fibrosis (DIF); **d**, the corresponding immunofluorescence analysis (*n* = 2, mean ± standard deviation, *p < 0.05 by t-test). The inset yellow text indicates the resultant vector lengths of each AoLD. NC normal control, Scale bars, 500 μm. α-SMA alpha-smooth muscle actin, COL1,type I collagen

Cell-induced fibrosis (CIF) was created by reversing the cell ratio of cardiac fibroblasts (CF) to cardiac myocytes (CM), recapitulating overproliferated CF in fibrosis. The MIR-DS-PAM results for CIF show that both the average PA amplitude and the AoLD uniformity were decreased (Fig. 4a), indicating reduced protein density and disrupted fiber alignment. Compared with immunofluorescence markers (Fig. 4b and Fig. S4), the CFM images corresponded well with the average PA MAPs; however, it is difficult to quantify the ECM alignment on a macroscale. The expression level of alpha-smooth muscle actin (α-SMA), a representative biomarker for myofibroblast activation (in fibrosis), was increased, and the expression of type I collagen (COL1) corresponded to the average PA amplitude pattern (white arrowheads). Likewise, drug-induced fibrosis (DIF) was triggered by applying the transforming growth factor-beta (TGF-β), a key cytokine that induces cardiac fibrosis. The MIR-DS-PAM results for DIF show an increased PA amplitude and the decreased AoLD uniformity (Fig. 4c), indicating extensive ECM accumulation but with disorganized orientation. Immunofluorescence analysis confirmed the upregulation of both α-SMA and COL1 (Fig. 4d).

Together, these results demonstrate that MIR-DS-PAM enables label-free and quantitative assessment of phenotypic differences in EHT fibrosis. Both CIF and DIF exhibited a subtle reduction in DoLD (from 0.15 to 0.11 in CIF; from 0.16 to 0.13 in DIF) and reduced AoLD uniformity (from 0.94 to 0.66 in CIF; from 0.95 to 0.69 in DIF), indicating ECM disorganization as a common hallmark of fibrosis. However, their biochemical profiles diverged: CIF showed low PA signals and COL1 expression, suggesting a late-stage fibrotic state characterized by completed cardiac fibroblast proliferation and diminished cellular activity, while DIF exhibited strong PA signals and high COL1 level, indicating extensive ECM deposition driven by TGF-β signaling^49^. This stage of disease progression was further indicated by mechanical dysfunctions associated with fibrosis. Both fibrotic models exhibited reduced contractility; however, CIF demonstrated a ~ 4.6-fold increase in Young’s modulus, whereas DIF showed a ~ 3-fold increase (Fig. S5). The decreased AoLD uniformity indicates that the structural alignment is disrupted, potentially weakening connectivity and interfering with mechanical functions. These findings highlight MIR-DS-PAM’s capability of simultaneously capturing the structural and molecular characteristics of fibrotic progression in EHTs.

### MIR-DS-PAM in assembled EHT

**Fig. 5:**
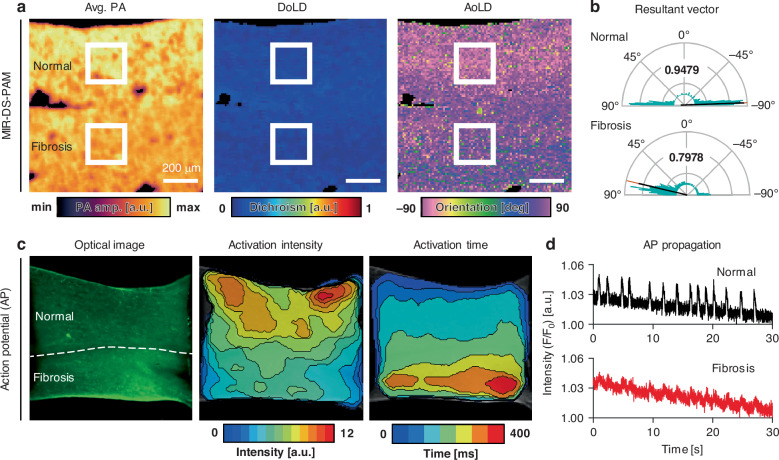
EHT assembly assessment. **a** MIR-DS-PAM images of assembled EHT. Scale bars, 200 μm. **b** Resultant vectors of orientation for the regions indicated by the white boxes in a. The inset bold text indicates the resultant vector length. **c** Action potential propagation analysis of the assembled EHT. The white dashed line indicates the border of the EHT. **d** Representative traces of normalized fluorescence signals (F/F_0_) in each EHT component, where F_0_ represents the baseline (resting) intensity

In life, tissues exist not as isolated units, but rather as spatially interconnected modules with structural coupling that is critical for proper function. To demonstrate MIR-DS-PAM’s ability to capture histostructural features in a composite tissue model, we extended the application to an assembled EHT. Normal and CIF EHT modules were assembled in parallel, and Fig. 5a shows representative MIR-DS-PAM images of the assembled EHT. As with the single CIF module seen earlier, the average PA signal amplitude is greater at the normal site than at the fibrotic site. The overall AoLD appears in the same direction as in single EHT modules. Notably, the fibrotic site exhibits a shorter resulting vector length than the normal site, implying EHT dysfunction in fibrosis (Fig. 5b). The decreased AoLD uniformity indicates that the structural alignment is disrupted, potentially weakening connectivity and interfering with mechanical functions. An action potential (AP) propagation analysis was conducted to validate the functionality of the normal and CIF EHT assembly (Fig. 5c and Fig. S6). APs were visualized using the FluoVolt™ membrane potential dye and recorded with a CMOS camera mounted on an inverted fluorescence microscope. APs generated in the normal EHT were delivered to the fibrotic EHT; however, the activation intensity was reduced, and the activation time was prolonged in the fibrotic tissue. In the abnormal EHT, the AP was delayed or even disappeared, identifying dysfunction (Fig. 5d). This assessment of a composite tissue assembly via MIR-DS-PAM demonstrates its potential for analyzing multicellular interactions and spatial heterogeneity within complex structures: MIR-DS-PAM reveals features that are not distinguishable through visual examination.

## Discussion

In this study, we present MIR-DS-PAM as a novel label-free dual-contrast imaging modality capable of protein-selective visualization and histostructural alignment investigation. By integrating MIR-PAM with polarization sensitivity, this platform leverages anisotropic optical absorption to simultaneously assess molecular composition and optical anisotropy within tissue samples. Unlike conventional histological or fluorescence-based modalities, MIR-DS-PAM requires no labeling and provides analytical histostructural information through its physically grounded quantitative approach. We validated the system in EHT models, demonstrating its ability to assess ECM changes during tissue development and disease modeling. The orientation uniformity, quantified by the resultant vector length of the AoLD, increased over five days, consistent with EHT maturation. Furthermore, MIR-DS-PAM-based histostructural analysis was demonstrated with two modeled phenotypes of EHT fibrosis, providing diagnostic cues from ECM organization.

Despite the promising functionalities of MIR-DS-PAM, several limitations remain. First, although MIR-DS-PAM provides intrinsic and quantitative imaging of ECM alignment, its spatial resolution is constrained by the diffraction limit of MIR wavelengths. By incorporating optical resolution enhancement techniques (e.g., structured illumination^50^ and synthetic aperture^51^) into the MIR regime, we expect not only to resolve subcellular structures but also to improve the precision of DS parameter calculations, enabling the analysis of single-fiber characteristics. In the axial direction, multilayer tissue analysis may also be achieved by adopting depth-of-focus extension techniques using diffractive optical elements^52^ and metalenses^53^. Additionally, several strategies for PA signal detection with high sensitivity can improve image quality and computational stability in DS-PAM^54–56^. Second, multiparametric analyses can lead to more accurate histological assessment. We have used a single wavelength for protein-selective imaging, capturing the main biomolecule associated with fibrous structures. Here, adopting multiple optical wavelengths can enable multispectral analysis to distinguish additional molecules in complex tissues^22,57^, such as other proteins, lipids, and cell nuclei. However, because the contrast of dichroism varies, appropriately designed polarizing components and systematic calibration are required to achieve a stable MIR-DS-PAM configuration across the operating wavelengths. By leveraging multidimensional spectral-polarization sensitivity^58^, MIR-DS-PAM is expected to further enhance the analytical capabilities of histopathological examination, particularly in scenarios where the molecular composition and structural organization change concurrently during tissue degeneration and regeneration. In addition, artificial intelligence-based techniques can be applied to interpret diagnostic cues^59^. Synthetic polarization parameter mapping can be achieved^60,61^, and automatic segmentation and classification on complicated datasets will facilitate more accurate histopathology^21^. Third, complex sample preparations and imaging conditions may introduce disturbing variability in amplitude-based DS metrics. Building on the label-free approach, a standardized protocol using slide-free volumetric imaging can mitigate interference to achieve reliable data acquisition and provide comprehensive histological examination. For 3D bioprinted organoids, MIR-DS-PAM enables label-free observation of structural and physiological functions^62,63^. Additionally, extending the modality’s application from in-vitro assessment to preclinical disease models and clinical biopsy samples would further enhance its translational relevance. MIR-DS-PAM can also establish structural-functional correlations with electrophysiological activity and be integrated with intracardiac echocardiography, forming a multimodal framework. Furthermore, histostructural analysis can be performed on highly birefringent tissues across various organ systems, including the heart, as well as the ocular and musculoskeletal systems^64–66^ (Fig. S7). These research directions will not only address current limitations but also broaden the applicability of MIR-DS-PAM as a dual-contrast functional imaging tool for regenerative medicine and pathology research.

## Materials and methods

### MIR-DS-PAM system

A nanosecond pulsed QCL (MIRcat, Daylight Solutions) was used as a light source for MIR-DS-PAM. The emitted beam was expanded and collimated using a lens pair (11–419 and 11–421, Edmund Optics). The linearly polarized light was controlled via a HWP (85–120, Edmund Optics) and partially reflected by an optical window (68–511, Edmund Optics) to monitor the laser power with a photodetector (PDAVJ10, Thorlabs). An objective lens with a numerical aperture of 0.52 (50102-02, Newport) focused light on the sample, and the generated PA signals were captured using an ultrasound transducer with a center frequency of 30 MHz. After the signals were passed through two amplifiers (ZFL-500LN, Mini-Circuits) and a low-pass filter (ZX75LP-40-S, Mini-Circuits), they were collected by a data acquisition board (NI PCIe-6321, National Instruments) with a digitizer (ATS9350, AlazarTech). LabVIEW software (LabVIEW 2017, National Instruments) was developed to control the XY stages (L-406.10SD00, Physik Instrumente) and a rotation stage (K10CR1, Thorlabs). For each pixel, 250 PA A-line signals were averaged, and the measured average laser power was about 0.26 mW.

### Cell culture

Cardiomyocytes were generated from WTC-11 human induced pluripotent stem cells (hiPSCs; GM25256, Coriell Institute) using a previously published protocol^4^. On day 2, hiPSCs were seeded at 250,000 cells per well on Matrigel-coated 12-well plates (356231, Corning) in mTeSR1 medium (ST85850, STEMCELL Technologies) supplemented with 10 μM Y-27632 (1254, Tocris Bioscience). On day 1, the medium was replaced with mTeSR1 supplemented with 1 μM CHIR99021 (13122, Cayman Chemical), and cells were cultured for 18 h. Cardiac differentiation medium was prepared using RPMI 1640 (11875-093, Thermo Fisher Scientific) supplemented with either B27 minus insulin (RPMI/B27-; A1895602, Thermo Fisher Scientific) or B27 with insulin (RPMI/B27 + ; 17504-044, Thermo Fisher Scientific). On day 0, the medium was replaced with RPMI/B27-containing 5 μM CHIR99021. On day 2, the cells were treated with RPMI/B27-containing 2 μM Wnt-C59 (S7037, Selleck Chemicals). On day 4, the medium was changed to RPMI/B27-only, and on day 6, it was replaced with RPMI/B27 + . Fresh RPMI/B27+ medium was provided every other day thereafter. Between days 12 and 16, differentiated cells were dissociated, and approximately 12 million cells were replated onto 100 mm dishes for lactate-based metabolic purification. The cells were cultured for 2–4 days in DMEM without glucose (11966025, Thermo Fisher Scientific) supplemented with 4 μM sodium L-lactate (L7022, Sigma Aldrich) and 1 mM sodium pyruvate (11360070, Thermo Fisher Scientific). After purification, the cells were maintained in RPMI/B27+ medium until use.

Human cardiac fibroblasts (CFs; C-12375, PromoCell) were cultured in Fibroblast Growth Medium (C-23130, PromoCell) and passaged every 2–3 days. Cells between passages 6 and 8 were used for engineered heart tissue (EHT) generation.

### Decellularization of extracellular matrix bioink preparation

We prepared hdECM powder according to previously described protocols^4^. Briefly, the left ventricular myocardium was extracted and minced into approximately 1 mm-thick pieces. Tissues were sequentially treated with tap water for 1 hour, 1% sodium dodecyl sulfate for 72 hours, 1% Triton X-100 for 1 h, 2-propanol for 2 h, and phosphate-buffered saline (PBS) for 72 hours, followed by 0.1% peracetic acid in 4% ethanol for 4 hours and extensive PBS rinses. After decellularization, the tissues were lyophilized, ground into powder using a mortar, and stored at −80 °C. The lyophilized dECM powder was digested in 0.5 M acetic acid and 10% (w/w) pepsin for 72 h. The resulting dECM solution was neutralized using 10 N NaOH and diluted with PBS to a final concentration of 10 mg/mL.

Cell-laden bioink was prepared by mixing iPSC-CMs and CFs at a 9:1 ratio in hdECM solution to a final cell density of 50 million cells/mL.

### Engineered heart tissue generation

Engineered heart tissues (EHTs) were fabricated using an extrusion-based bioprinting system (3DXPrinter, T&R Biofab), as previously reported^4^. Briefly, a polyethylene vinyl acetate (PEVA) construct consisting of a frame and two anchoring pins was fabricated. Cell-laden hdECM bioink was then extruded onto the PEVA construct, and the EHTs were incubated at 37 °C for 60–90 min to promote thermal crosslinking. The printed EHTs were cultured in RPMI/B27+ medium supplemented with 10% FBS and 10 μM Y-27632 for the first 24 h. Thereafter, the culture was continued in RPMI/B27+ medium with 1% FBS.

For EHT assembly, the PEVA frames were carefully removed, and the EHTs were transferred to the EHT assembly platform. The anchoring pins were manually inserted into designated holes in the assembly platform, using forceps.

### Fibrosis modeling of engineered heart tissue

Two strategies were employed to generate fibrotic EHTs. Cell-induced fibrosis (CIF) was established by inverting the iPSC-CM:CF ratio to 1:9. Drug-induced fibrosis (DIF) was achieved by treating the EHTs with 20 ng/ml TGF-β for 2 weeks.

### Immunofluorescence staining analysis

EHT samples were embedded in paraffin blocks, sectioned, and mounted on ZnSe windows or glass slides for imaging. For immunofluorescence staining, the samples were deparaffinized using xylene and rehydrated through a graded ethanol series. Antigen retrieval was performed by immersing the samples in citrate buffer, heating them in a microwave until boiling, and incubating at room temperature for 1 h. Afterwards, the samples were permeabilized with 0.1% triton X-100 solution for 10 min and blocked with 5% normal goat serum for 1 h. Primary antibodies were incubated at 4 °C for 16 h, followed by 2 h of incubation with fluorescent secondary antibodies at room temperature. The antibodies used in this experiment are listed in the Supplementary Table S2. Fluorescence imaging was performed using a spinning disk confocal microscope (CSU-W1 SoRa, Nikon).

### Contractile force and Young’s modulus measurement

The mechanical characteristics of the EHTs were assessed using an Isolated Muscle System (1500 A, Aurora Scientific). EHTs were incubated in Tyrode’s solution at 37 °C for 15 min prior to testing. After the frame structure was removed, the EHTs were mounted onto the force measurement system. The assessment was conducted in Tyrode’s solution at 37 °C following standardization of the initial tissue length. To determine the Young’s modulus, stepwise stretching was applied, and the corresponding forces were recorded. The cross-sectional area of each EHT was measured after testing and used for the calculation of the modulus.

### Action potential propagation assessment

Normal and fibrosis EHTs were assembled in parallel according to a previously established protocol^4,49^ and used to assess action potentials (APs) to demonstrate compartmentalized electrophysiological properties. EHTs were stained using the FluoVolt™ Membrane Potential Kit (F10488, Thermo Fisher Scientific). Samples were washed twice with Tyrode’s solution, then incubated with the FluoVolt loading solution for 30 min at room temperature in the dark. Following incubation, the FluoVolt loading solution was removed, and the samples were washed twice with Tyrode’s solution. During imaging, samples were immersed in fresh Tyrode’s solution and maintained at 37 °C. AP propagation was recorded at 100 frames per second using an ORCA-Flash4.0 V3 Digital CMOS camera (C13440-2CU, Hamamatsu) mounted on an inverted fluorescence microscope (ECLIPSE Ti, Nikon), with an exposure time of 5 ms. A single cycle of activation was optically mapped using custom-made MATLAB software. AP intensity was quantified as the ratio of fluorescence intensity (F) to the baseline (resting) intensity (F₀).

## Supplementary information

Supplementary Information

## Data Availability

The data that support the findings of this study are available from the corresponding author upon reasonable request.
