# Supplementary material for: Label-free mid-infrared dichroism-sensitive photoacoustic microscopy for histostructural analysis of engineered heart tissues
Source: Light Sci Appl. 2026 Jan 4;15:49. doi: 10.1038/s41377-025-02117-0 (PMC12764944; doi:10.1038/s41377-025-02117-0)
Supplement: Supplementary file 1 — Supplementary Information [file 41377_2025_2117_MOESM1_ESM.pdf]

Supplementary information for:

Label-free mid-infrared dichroism-sensitive photoacoustic microscopy for histostructural analysis of engineered heart tissues

Eunwoo Park[1,2,§], Dong Gyu Hwang[2,3,§], Hwanyong Choi[2,4], Donggyu Kim[1,2], Joongho Ahn[2,5,6], Yong-Jae Lee[7], Tae Joong Eom[7,8], Jinah Jang[1,2,3,4,9,10,*], and Chulhong Kim[1,2,4,5,6,9,11,*]

[1]*Department of Convergence IT Engineering, Pohang University of Science and Technology (POSTECH), Pohang, Republic of Korea*

[2]*Medical Device Innovation Center, Pohang University of Science and Technology (POSTECH), Pohang, Republic of Korea*

[3]*Center for 3D Organ Printing and Stem Cells, Pohang University of Science and Technology (POSTECH), Pohang, Republic of Korea*

[4]*Department of Mechanical Engineering, Pohang University of Science and Technology (POSTECH), Pohang, Republic of Korea*

[5]*Department of Electrical Engineering, Pohang University of Science and Technology (POSTECH), Pohang, Republic of Korea*

[6]*Opticho Inc., Pohang, Republic of Korea*

[7]*Engineering Research Center for Color-modulated Extra-sensory Perception Technology, Pusan National University, Busan, Republic of Korea*

[8]*Department of Congo-Mechatronics Engineering & Optics and Mechatronics Engineering, Pusan National University, Busan, Republic of Korea*

[9]*Department of Medical Science and Engineering, Pohang University of Science and Technology (POSTECH), Pohang, Republic of Korea*

[10]*Institute for Convergence Research and Education in Advanced Technology, Yonsei University, Seoul, Republic of Korea*

[11]*Graduate School of Artificial Intelligence, Pohang University of Science and Technology (POSTECH), Pohang, Republic of Korea*

[§]These authors contributed equally to this work: Eunwoo Park and Dong Gyu Hwang

[*]Corresponding authors: Chulhong Kim (chulhong@postech.edu) and Jinah Jang (jinahjang@postech.ac.kr)

Table of Contents

Supplementary Notes

Note. S1. Immunofluorescence staining in fibrotic EHT 5

Supplementary Figures

Fig. S1. PS-OCT images in ultrathin samples 2
Fig. S2. MIR-DS-PAM verification using nylon threads 3
Fig. S3. Orientation vector annotations on AoLD 4
Fig. S4. Immunofluorescence markers in fibrotic EHT 5
Fig. S5. Mechanical characterization of fibrotic EHTs 6
Fig. S6. Action potential propagation in assembled EHTs 7
Fig. S7. MIR-DS-PAM in *ex vivo* mouse leg 8

Supplementary Tables

Table. S1. MIR-DS-PAM EHT assessment by the day 4
Table. S2. List of antibodies used for immunofluorescence staining 9

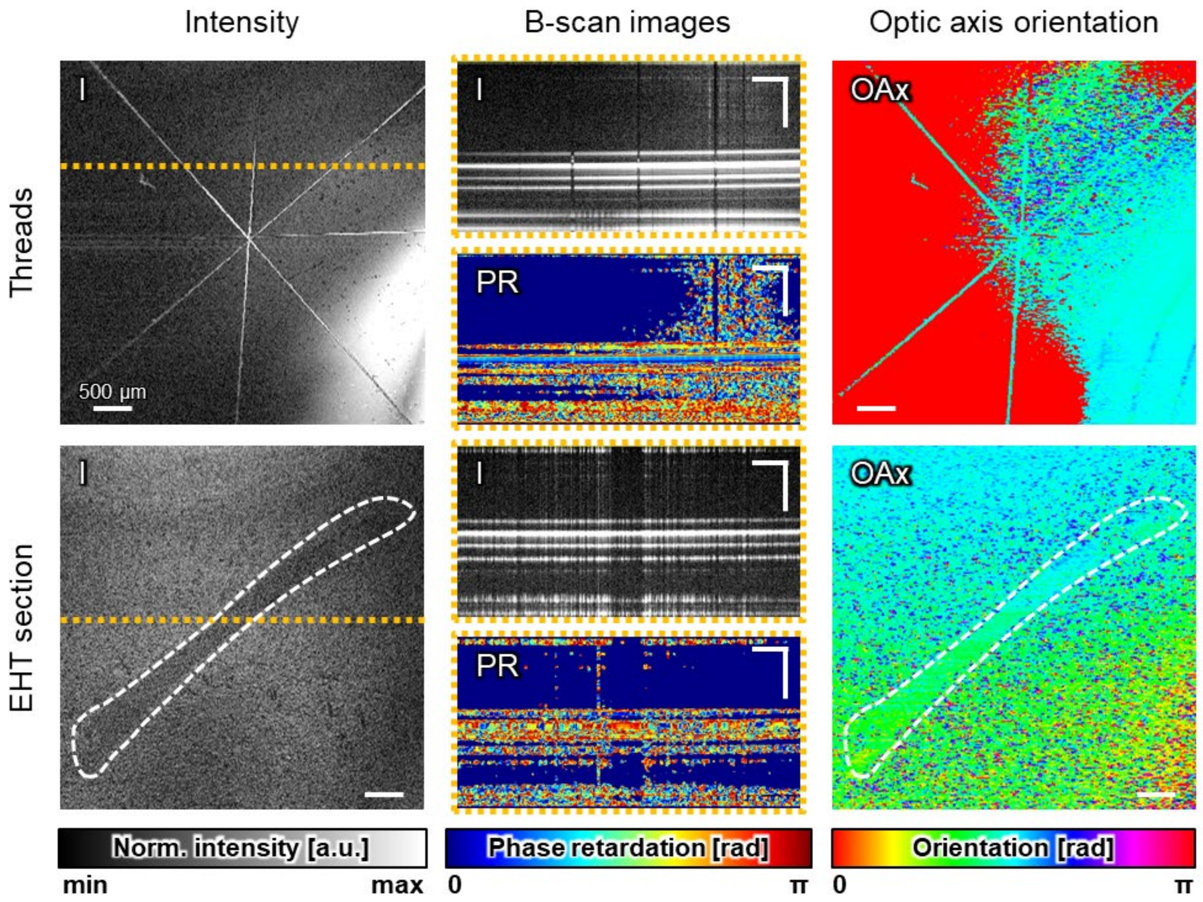

Fig. S1. Polarization-sensitive optical coherence tomography (PS-OCT) images in ultrathin samples[1]. I, intensity; PR, phase retardation; OAx, optic axis orientation. Scale bars, 500 µm. B-scan images represent the cross-sectional plane indicated by the orange dotted lines in the corresponding *en-face* intensity image. In ultrathin sections, strong surface reflections, low backscattering signal, and insufficient phase retardation lead to degraded PS-OCT contrast.

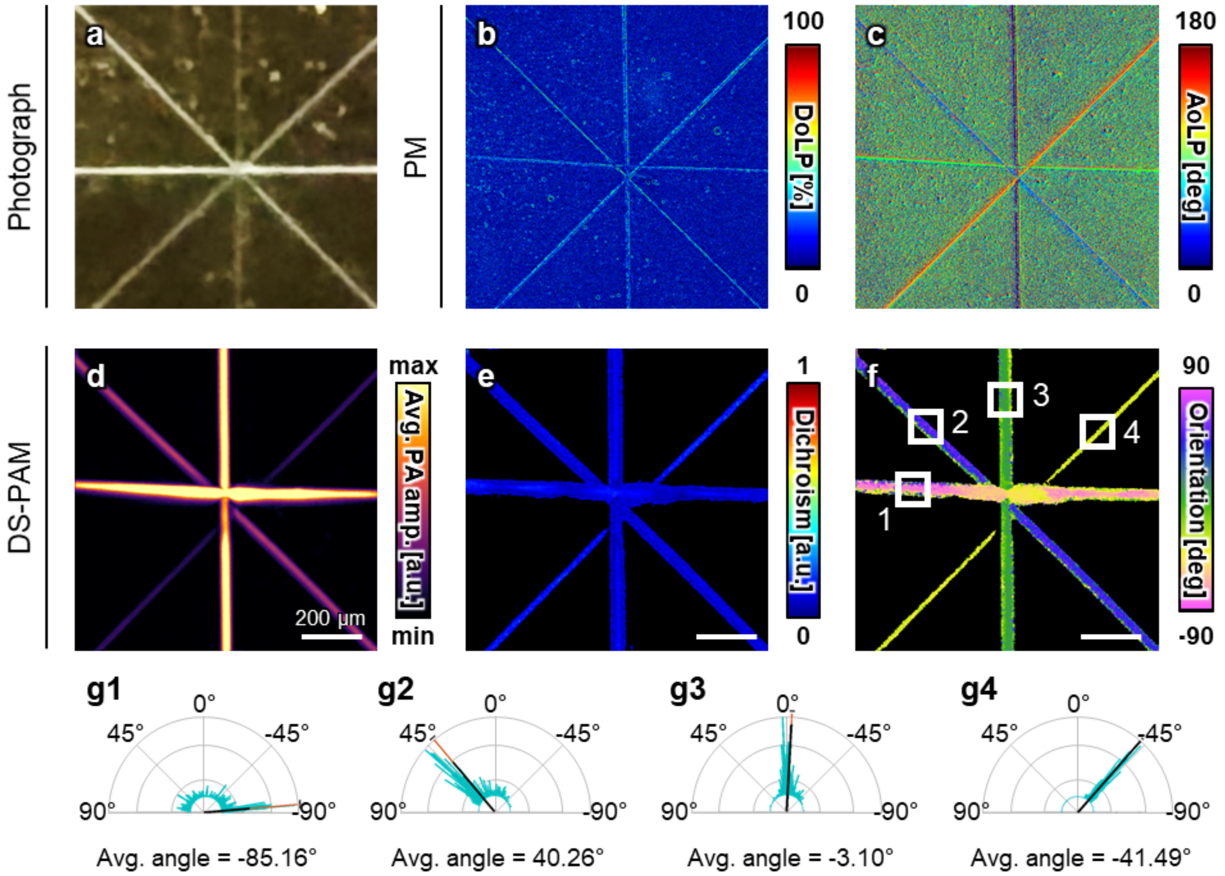

Fig. S2. MIR-DS-PAM verification using polyamide monofilament threads. a, Photograph of intersecting threads. **b–c,** Polarization microscopy (PM) images: degree and angle of linear polarization (DoLP and AoLP, respectively). **d–f,** MIR-DS-PAM images: average PA MAP, DoLD, and AoLD, respectively. Scale bars, 200 µm. **g1–g4,** AoLD polar histograms of each region in the numbered white boxes in **f**. The average angle of the corresponding threads is noted below each plot.

Table S1. MIR-DS-PAM EHT assessment by the day. All data are presented as mean ± standard deviation.

	Day 1	Day 2	Day 3	Day 4	Day 5
Avg. PA [mV]	11.89±2.40	12.47±1.91	15.97±1.95	15.27±2.19	19.39±2.50
DoLD [a.u.]	0.14±0.05	0.15±0.04	0.16±0.04	0.17±0.05	0.19±0.04
AoLD [deg]	33.45±11.62	16.88±9.16	36.12±8.40	-53.52±7.81	-37.28±6.09

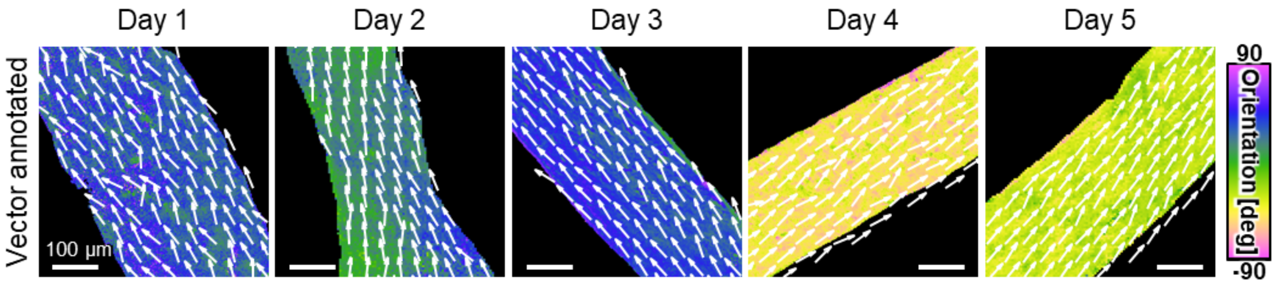

Fig. S3. Orientation vector annotation on AoLD. Local orientation was visualized by annotating vectors (white arrows) at corresponding positions on the AoLD map. Scale bars, 100 µm

Supplementary Note 1. Immunofluorescence staining in fibrotic EHT

Quantification of fluorescent intensity confirmed that both CIF and DIF fibrotic EHT models display cardiac fibrosis-like cellular and ECM changes. Vimentin, a fibroblast marker, was increased in both the CIF and DIF models, because of increased fibroblast proportions and proliferation after drug treatment, respectively. In addition, alpha-smooth muscle actin (α-SMA), a marker expressed in activated fibroblasts in fibrotic environments, also increased in both models. Type I collagen (COL1) decreased in CIF, whereas it increased in DIF, consistent with MIR-DS-PAM sensitivity findings. In CIF, it can be inferred that overpopulated fibroblasts suppressed the activation of cells, whereas in DIF, cells remodeled ECM composition in response to TGF-β signaling, resulting in increased collagen deposition[2]. The increases in the expression of DAPI may be attributed to the increased numbers and sizes of cell nuclei.

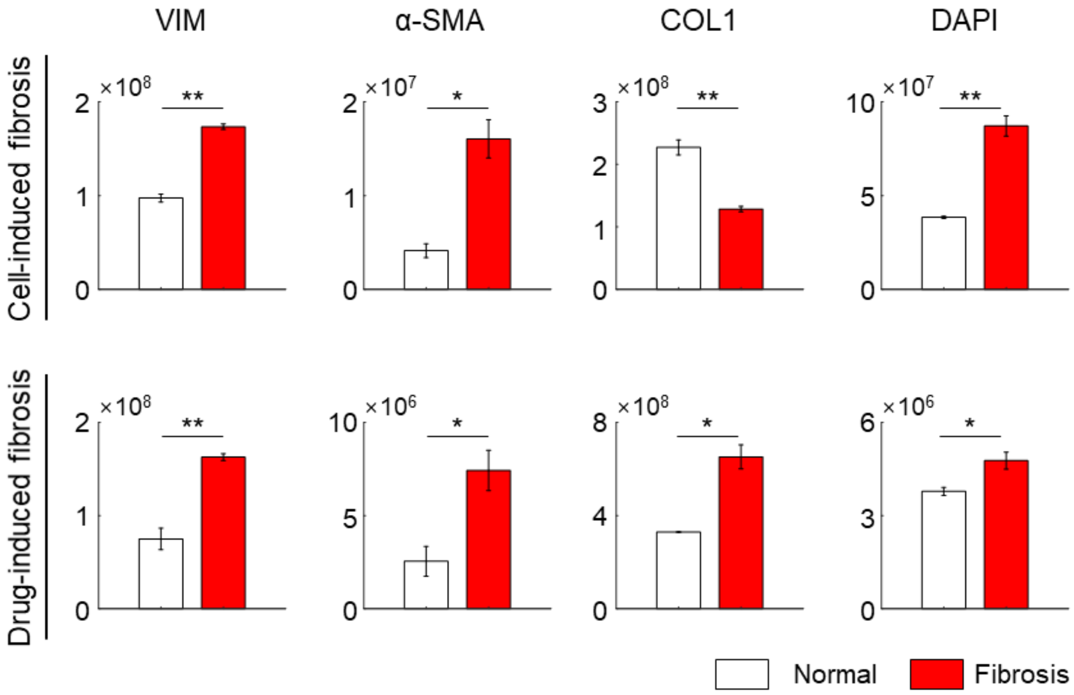

Fig. S4. Immunofluorescence markers in fibrotic EHT. VIM, vimentin; α-SMA, alpha-smooth muscle actin; COL1, collagen type 1 (n = 2, mean ± standard deviation, **p* < 0.05, ***p* < 0.01 by t-test).

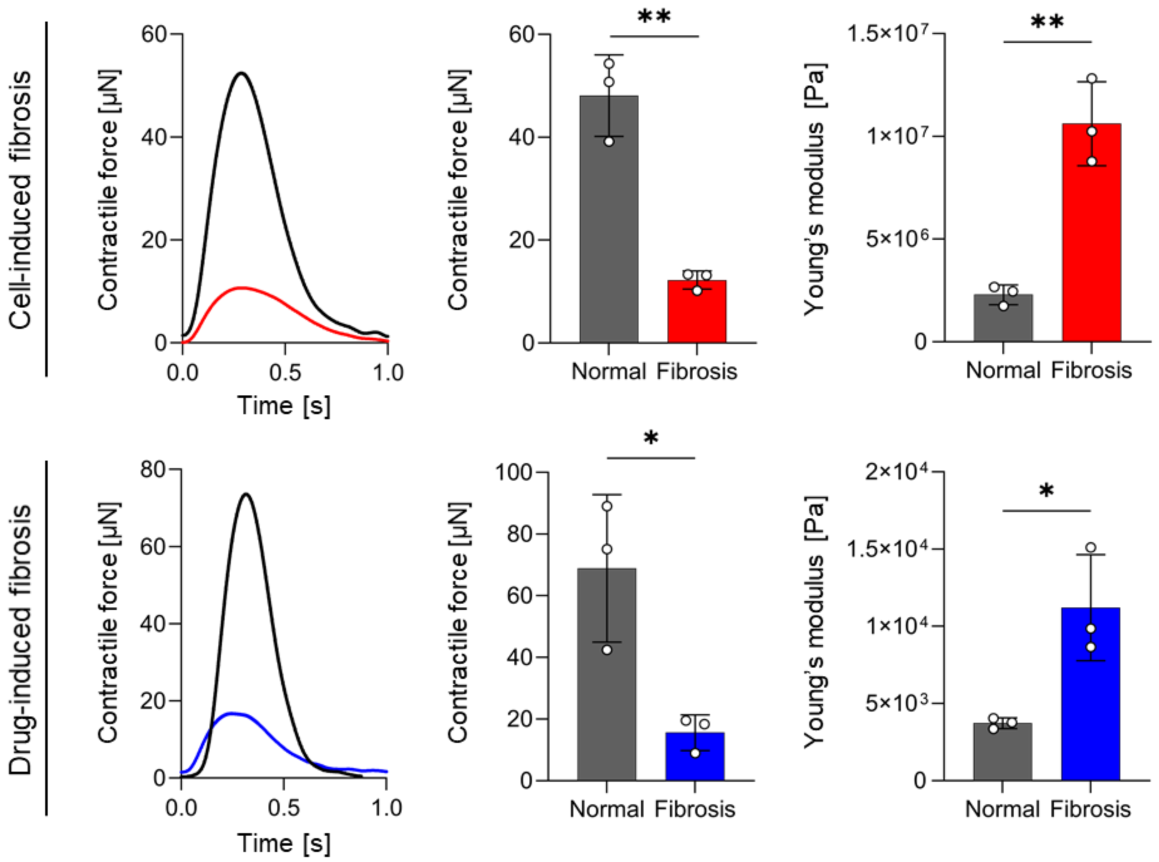

Fig. S5. Mechanical characterization of fibrotic EHTs. (n = 3, mean ± standard deviation, *$p < 0.05$, **$p < 0.01$)

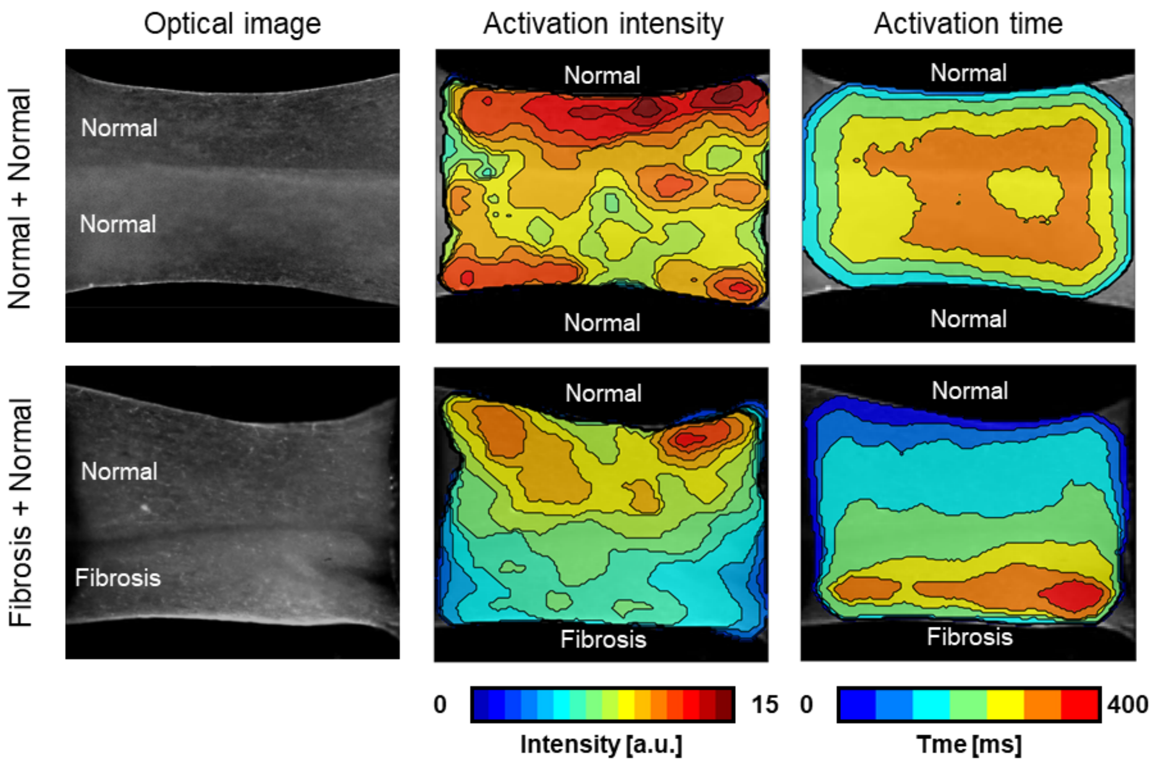

Fig. S6. Action potential propagation in assembled EHTs. (upper row) Normal-normal EHT assembly. (lower row) Normal-fibrosis EHT assembly.

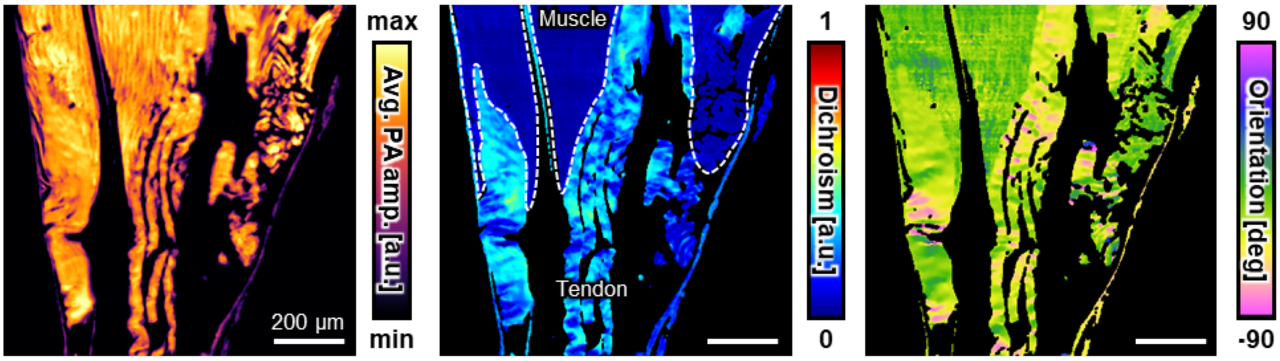

Fig. S7. MIR-DS-PAM of an *ex vivo* mouse leg. The triceps surae muscle and Achilles tendon are visualized by protein-selective MIR-PAM. High DoLD is observed in the mouse Achilles tendon due to the high proportion of type I collagen, which exhibits high birefringence.

Table S2. Antibodies used for immunofluorescence staining.

Antibody	Catalog #	Dilution
Anti-sarcomeric alpha actinin	ab9465; Abcam	1:200
Anti-vimentin	ab24525; Abcam	1:200
Anti-collagen I	ab6308; Abcam	1:200
Fluorescein phalloidin	F432; Thermo Fisher Scientific	1:40
Goat-anti-Mouse secondary AF488	A-11001; Thermo Fisher Scientific	1:200
Goat-anti-Rabbit secondary AF594	A-11012; Thermo Fisher Scientific	1:200
Goat-anti-Chicken secondary AF405	ab175674; Abcam	1:200

References

1 Lee, Y. J. *et al.* Quantification method to objectively evaluate the fibrous structural status of tendons based on polarization-sensitive OCT. *Journal of Biophotonics* **15**, e202200065 (2022). https://doi.org:10.1002/jbio.202200065

2 Hwang, D. G. *et al.* Bioprinting-Assisted Tissue Assembly to Investigate Endothelial Cell Contributions in Cardiac Fibrosis and Focal Fibrosis Modeling. *Advanced NanoBiomed Research*, 2400148 (2025). https://doi.org:10.1002/anbr.202400148
